# Differential expression in genes involved in the NF-κβ pathway among asymptomatic HTLV-1 carriers and HAM/TSP patients in Peru

**DOI:** 10.1186/1742-4690-11-S1-P126

**Published:** 2014-01-07

**Authors:** Michael Talledo, Jason Rosado, Carolina Alvarez, Daniel Clark, Eduardo Gotuzzo

**Affiliations:** 1 Laboratorio de Epidemiología Molecular, Universidad Peruana Cayetano Heredia, Lima, Peru; 2 Instituto de Medicina Tropical Alexander von Humboldt, Universidad Peruana Cayetano Heredia, Lima, Peru; 3 Rega Institute for Medical Research, Katholieke Universiteit Leuven, Leuven, Belgium; 4 Laboratorios de Investigación y Desarrollo, Universidad Peruana Cayetano Heredia, Lima, Peru; 5 Facultad de Medicina, Universidad Peruana Cayetano Heredia, Lima, Peru; 6 Hospital Nacional Cayetano Heredia, Lima, Peru

## 

HTLV-1-associated myelopathy/tropical spastic paraparesis (HAM/TSP) is a neuroinflammatory, non-remitting and disabling disease. NF-κβ pathway plays a role in the pathogenesis of this condition. We compare the expression at peripheral level of84 genes involved in the NF-κβ pathway among asymptomatic HTLV-1 carriers (AC) and HAM/TSP patients. mRNA from PBMCs was isolated from 12 HTLV-1 carriers classified into three groups: fourAC (=patients without any neurological condition) and eight patients with HAM/TSP (four with EDSS scores of 1-5 and four with EDSS score of 5.5-9.0). 84 genes related to the NF-κβ pathway were evaluated using Superarray plates (SABiosciences) and Real Time PCR in both groups. Ct values under 35 were considered positive, False Discovery Rate was used to control by multiple comparisons. Three genes were dysregulated in HAM/TSP patients. The expression level of NF-κβIA was higher in AC compared to HAM/TSP patients, while EGR1 and IL-8 showed a lower expression in AC compared to HAM/TSP patients (p<0.05 after multiple testing correction). These results are in agreement to our previous genetic findings; nevertheless, a validation in an independent group is required to confirm these results. Further studies evaluating the role of genes involved in the NF-κβ pathway as potential biomarkers for HAM/TSP are needed.

